# Identification and interaction analysis of hubgenes related to neutrophil ferroptosis in intracranial atherosclerotic stenosis

**DOI:** 10.1590/1678-4685-GMB-2024-0106

**Published:** 2025-11-17

**Authors:** Yilin Wang, Tao Wang, Ziping Han, Rongliang Wang, Yue Hu, Zhenhong Yang, Tong Shen, Yangmin Zheng, Jichang Luo, Yan Ma, Yumin Luo, Liqun Jiao

**Affiliations:** 1 Capital Medical University, Xuanwu Hospital, Institute of Cerebrovascular Disease Research, Department of Neurology, Beijing, China.; 2 Capital Medical University, Beijing Anzhen Hospital, Neurological Disease Center, Cerebrovascular Diseases Inpatient Ward 1, Beijing, China.; 3 Capital Medical University, Xuanwu Hospital, Department of Neurosurgery, Beijing, China; 4 Beijing Geriatric Medical Research Center, Beijing Key Laboratory of Translational Medicine for Cerebrovascular Diseases, Beijing, China.; 5 Capital Medical University, Beijing Institute for Brain Disorders, Beijing, China.

**Keywords:** Atherosclerosis, ferroptosis, neutrophil, stroke

## Abstract

Circulating neutrophils and ferroptosis are vital for the development of intracranial atherosclerotic stenosis (ICAS). This study aimed to explore that whether neutrophil ferroptosis participate in ICAS. Sixteen patients with ICAS and 14 healthy controls were enrolled. We collected peripheral blood and separated neutrophils. LncRNA, mRNA, and miRNA sequencing were performed. The differently expressed (DE) lncRNAs, mRNAs, and miRNAs were selected. The protein-protein interaction (PPI) network was constructed, top 30 hubgenes were selected, and intersected with DE ferroptosis genes to obtain the core genes. Combined with DE lncRNAs and miRNAs, the ceRNA network and TF-miRNA-mRNA network were constructed. Finally, the expression levels of the core genes were verified by Quantitative Reverse Transcription PCR (qRT-PCR) in other 11 patients and 9 healthy controls. The five core ferroptosis hubgenes were preliminarily identified, and ceRNA network and TF-miRNA-mRNA network were constructed. We identified several pairs of interactions. Finally, verification of qRT-PCR results revealed that CTSB (P=0.001) and HNRNPL (P=0.002) were upregulated, KRAS (P=0.003) and MAP1LC3A (P=0.004) were downregulated in ICAS group, compared with healthy controls. Our results identified four core genes (CTSB, HNRNPL, KRAS and MAP1LC3A) and constructed potential regulatory network, providing the potential therapeutic targets for ICAS.

## Introduction

Intracranial atherosclerotic stenosis (ICAS) is a main cause of stroke and recurrent stroke. In Asia, about 50 % of strokes are attributed to ICAS (Chaturvedi, 2020; Kalirawna et al., 2021; Gutierrez et al., 2022). To date, it has been increasingly recognized that the asymptomatic patients with ICAS are exposed to silent cerebral infarction and dementia (Gutierrez *et al*., 2022). It is well known that atherosclerosis belongs to a chronic inflammatory vascular disease (Bir and Kelley, 2022). As a classical member of inflammatory family, neutrophils fulfill various roles in atherosclerosis during its initiation and progression, such as aggravating endothelial dysfunction and activating macrophages (Geng et al., 2019). Despite the importance of neutrophils in atherosclerosis, neutrophils are rarely detected in atherosclerotic plaques, which may be attributed to its short life span and accordingly, macrophages can rapidly remove the senescent neutrophils in the developing plaque (Gomez et al., 2020). Ferroptosis has been considered as one of the most ancient and widespread type of cell deaths, caused by iron-dependently lethal lipid peroxidation. Ferroptosis can be attributed to redox homeostasis imbalance and cellular metabolism, which can be suppressed by iron depletion or lipid peroxidation blockade (Jiang et al., 2021). Literature has reported that ferroptosis may participate in the initiation and development of atherosclerosis (AS). Imbalanced intracellular iron can damage macrophages, smooth muscle cells and endothelial cells. Meanwhile, ferroptosis correlates with AS-related pathophysiological processes, such as inflammation, lipid peroxidation and oxidative stress (Ouyang et al., 2021). Compared with atherosclerotic plaques, iron accumulation is significantly higher than that in healthy arteries (Li *et al*., 2023). Elevated intracellular iron accumulation may facilitate inflammation reaction, lipid oxidation, and necrotic cores, increasing the vulnerability of atherosclerotic plaques and occurrence of life-threatening vascular diseases (Gao et al., 2023; Li *et al*., 2023). Despite the importance of circulating neutrophils and ferroptosis in atherosclerosis, the role that ferroptosis of circulating neutrophils play in ICAS is not reported previously. Non-coding RNAs have been found to play an important role in several physiological and pathological cellular processes (such as differentiation, proliferation, senescence, apoptosis, and ferroptosis) and be responsible for gene expression and signaling pathways related to various diseases (Mou et al., 2019; Elgebaly et al., 2021 b, 2023). We identified several core genes related to neutrophil ferroptosis and constructed potential regulatory network, such as protein-protein interaction (PPI) and ceRNA network (Elgebaly *et al.*, 2021a), aiming to provide insights into the potential pathogenesis and therapeutic targets of ICAS, from the perspective of neutrophil ferroptosis. Firstly, we identified differentially expressed (DE) mRNAs between ICAS and control group, constructed protein-protein interaction (PPI) network, selected top 30 hubgenes, and intersected with ferroptosis genes. Finally, five core genes were obtained. Previous literature has reported that CTSB can facilitate lipid deposition and increase plaque area (Qiu et al., 2025). KRAS participates in the formation of atherosclerotic plaques (Ban et al., 2024). ALB has been identified as an important anti-atherosclerosis targets (Yuan et al., 2022). MAP1LC3A is involved in endothelial dysfunction by participating in autophagy, participating in atherosclerosis process (Yamagata et al., 2015). Regarding the relationship between HNRNPL and atherosclerosis, there is no literature available.

## Material and Methods

### Patients inclusion and neutrophils isolation

The Ethics Committee of the Xuanwu Hospital of Capital Medical University granted ethical approval for this study (No. [2021]083 in October 2021) and written informed consent was obtained from all enrolled individuals at enrollment. The study conforms to the Declaration of Helsinki. The patients admitted to Xuanwu Hospital and diagnosed with ICAS via a high-resolution MRI were enrolled in our study. We mainly focus on identifying the potential mechanism of intracranial atherosclerotic stenosis. Given that the patients with other severe diseases may have a history of long-term drug intake, both the disease itself and the drug-intake may intervene the natural pathological process. Therefore, the exclusion criteria were listed as follows: (1) severe organic diseases, (2) other system diseases, (3) a history of other neurological disorders such as stroke and brain trauma, (4) previous history of long-term drug intake, such as antiplatelet drugs, (5) incomplete clinical data. Totally, 16 patients and 14 healthy volunteers were enrolled in our study. Neutrophils were separated and purified from the peripheral blood of all the participants within 3 hours via density gradient centrifugation, as previously described (Ma et al., 2019). Firstly, plasma was isolated from the peripheral blood samples via centrifugation at 200×*g* for 10 min. Subsequently, mononuclear cells were isolated via centrifugation at 400×*g* for 20 min. And then, the remaining part was mixed with homemade erythrocyte lysis buffer and centrifuged at 3000 × r for 10 min to lyse erythrocyte. Afterwards, the mixture was centrifuged at 3000×*g* for 10 min to isolate neutrophils. Finally, neutrophils total RNA extraction was performed via TRIzol reagent. Preliminary microarrays were performed on 5 patients and 5 healthy controls. Further verification by Quantitative Reverse Transcription PCR (qRT-PCR) were performed on the remaining 11 patients and 9 healthy controls. 

### RNA extraction, LncRNA, mRNA and miRNA microarrays

Total RNAs were extracted from the neutrophils via TRIzol reagent (Invitrogen, Carlsbad, CA, USA) and quality and quantity evaluations of the extracted RNAs were performed via NanoDrop ND-1000. The human 4 × 180 K lncRNA, mRNA, and miRNA microarrays (Arraystar Human LncRNA Microarray v4.0) were performed using a Gene Expression Hybridization Kit (Agilent, Santa Clara, CA, US). Based on the random priming method (Arraystar Flash RNA Labeling Kit, Arraystar), mRNA was purified, amplified and labeled. To fragment the labeled cRNA, blocking agent and fragmentation buffer were added, and then the mixture was heated at 60 °C for 30 min. The diluted hybridization solution was dispensed into a gasket slide, which was assembled to the lncRNA, mRNA and miRNA expression microarray slide. Slides were washed and scanned by a Gene Expression Wash Buffer Kit (Agilent, Santa Clara, CA, USA) and an Agilent Microarray Scanner (Agilent, Santa Clara, CA, USA), respectively. 

### Identification of differentially expressed (DE) lncRNAs, DE miRNAs, DE mRNAs, and the core genes

The raw data were extracted from scanned images. Gene Spring Software 12.6 (Agilent Technologies) was adopted for quantile normalization of raw data. R software (version 4.1.0) with the *samr* package was adopted for identifying the differentially expressed (DE) lncRNAs, mRNAs and miRNAs. LncRNA, mRNA and miRNA expression profiling of ICAS patients was obtained by calculating the fold change (FC) against the average of the healthy controls. LncRNAs, mRNAs or miRNAs with FC > 2.00 and P values < 0.05 were identified as the DE ones. Based on the DE mRNAs, the protein-protein interaction (PPI) network was constructed via the online database STRING (Search Tool for the retrieval of Interacting Genes/Proteins) and Cytoscape 3.9.1., and then top 30 hubgenes were selected via Cytohubba plugin. Ferroptosis gene list was downloaded on the Genecards database. DE mRNAs and ferroptosis gene list were intersected to obtain the genes that were differently expressed between two groups and related to ferroptosis (DE ferroptosis genes). Gene Ontology (GO) enrichment analysis and Kyoto Encyclopedia of Genes and Genomes (KEGG) pathway analysis were performed for these genes via Database for Annotation, Visualization, and Integrated Discovery (DAVID). The GO terms consist of biological process (BP), molecular function (MF) and cellular component (CC). Top 30 hubgenes and DE ferroptosis genes were intersected to obtain the core ferroptosis hubgenes. The detailed process is shown in Figure S1. 

### Construction of ceRNA network and TF-miRNA-mRNA network

We constructed ceRNA network and TF-miRNA-mRNA network for the core ferroptosis hubgenes. Regarding the ceRNA network, lncRNA-miRNA and miRNA-mRNA interaction were predicted by TargetScan and miRanda databases. The predicted lncRNAs and miRNAs were intersected with DE lncRNAs and DE mRNAs, and then the intersected lncRNAs and miRNAs were put into the preliminary ceRNA network. To simplify the network, the binding sites of miRNA-mRNA were predicted by TargetScan and ENCORI databases. The verified miRNA-mRNA pairs and their corresponding lncRNAs were extracted from the preliminary network and then put into the simplified network. Regarding the TF-miRNA-mRNA network, mRNA-TF, mRNA-miRNA and miRNA-TF interaction were predicted via Animal TF3.0, ENCORI and TrandMiR2.0 databases. Predicted miRNAs were intersected with DE miRNAs, and mRNA-predicted TFs were intersected with miRNA-predicted TFs. The intersected miRNAs and TFs were put into the preliminary network. To simplify the network, all the TFs in the network were intersected with the ferroptosis gene list. Finally, 9 TFs and related miRNAs put into the final network. The binding sites of the mRNA-TF were predicted via JASPAR database and visualized. The detailed process was shown in Figure S1. 

### Verification by qRT-PCR

Preliminary verification was performed by qRT-PCR. Total RNAs were extracted by TRIzol reagent, and RNA was reverse-transcribed into cDNA by Super Script III reverse transcriptase (Invitrogen). And then mRNA expression levels were calculated by Real-time PCR System using appropriate primers. The sequences of primers are displayed in Table S1. RTqPCR amplification was implemented via 2xPCR master mix on ViiA 7 Real-time PCR System (Applied Biosystems). β-actin was used as internal parameters to normalize RNA preparation. Relative mRNAs expression was calculated by the delta-delta Ct (ΔΔCt) method and then transformed to the relative expression ratio (2^-ΔΔCt^) for data analysis. To assess the amplification specificity, we constructed (range from 60 °C to 95 °C) a dissociation curve. The results are displayed as the FC in mRNA expression levels. 

### Statistical analysis and graph plotting

Significance of differences between the ICAS group and healthy controls were evaluated via the Mann-Whitney U test and P<0.05 was considered statistically significant. Statistical analyses were performed by SPSS Statistics 23.0 software (IBM Corp.). Graphs were generated by GraphPad prism 8.0 software, R software (version 4.4.1), Cytoscape 3.9.1, Ad obe Illustrator 2020 and an online platform (https://www.bioinformatics.com.cn).

## Results

### Identification of DE lncRNAs, mRNAs and miRNAs

The expression profiles of lncRNAs, mRNAs and miRNAs are visualized using volcano plots (Figure S2 A, B, C). Based on the filtering criteria (fold change>2 and P<0.05), DE lncRNAs, mRNAs and miRNAs in neutrophils were identified. And then heatmaps were created to display the top 50 DE lncRNAs, mRNAs and miRNAs (Figure S2 D, E, F).

### Identification of the DE ferroptosis genes and core genes, functional enrichment annotation

**Figure 1-:**
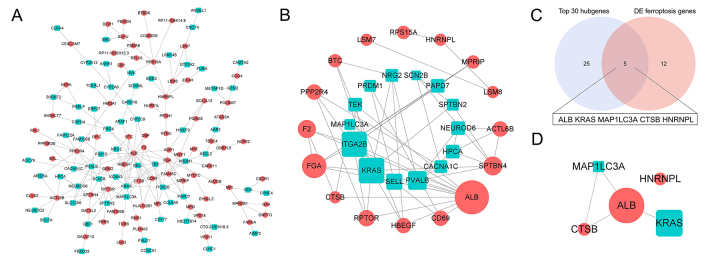
Identification of the core genes. (A) Protein-protein interaction network of differentially expressed mRNAs between intracranial atherosclerotic stenosis group and controls. Proteins and the interactions are visualized as nodes and edges, respectively. Red means upregulated mRNAs; green means downregulated mRNAs. (B) Top 30 nodes ranked by Degree algorithm calculated by Cytohubba plugin in Cytoscape software were extracted from the protein-protein interaction network. Sizes of nodes indicate the importance degree of the mRNAs in the network. (C) Top 30 hubgenes intersected with differentially expressed ferroptosis genes to obtain five core genes. (D) The 5 core genes extracted from protein-protein interaction network.

**Figure 2 -:**
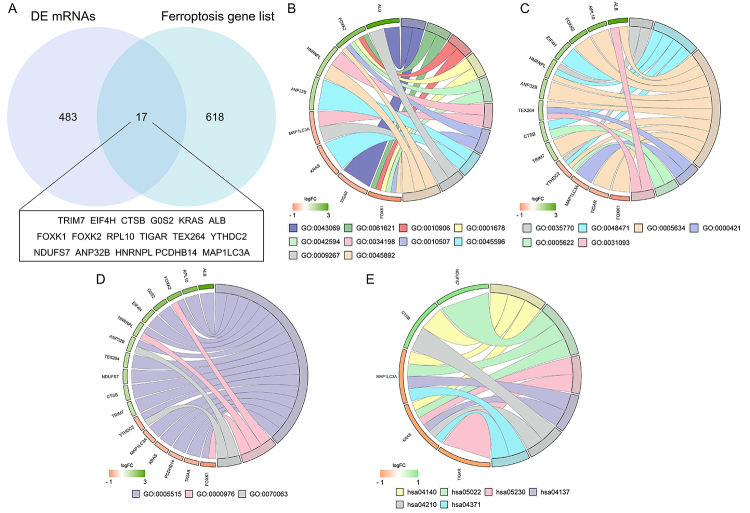
Identification of differentially expressed ferroptosis genes and functional enrichment annotation. (A) Differentially expressed mRNAs intersected with ferroptosis gene list to obtain 17 differentially expressed ferroptosis genes. DE: differentially expressed. (B) Gene Ontology analysis (biological process) of 17 genes. 0043069: negative regulation of programmed cell death; 0061621: canonical glycolysis; 0010906: regulation of glucose metabolic process; 0001678: cellular glucose homeostasis; 0042594: response to starvation; 0034198: cellular response to amino acid starvation; 0010507: negative regulation of autophagy; 0045596: negative regulation of cell differentiation; 0009267: cellular response to starvation; 0045892: negative regulation of transcription, DNA-templated. (C) Gene Ontology analysis (cellular component) of 17 genes. 0035770: ribonucleoprotein granule; 0048471: perinuclear region of cytoplasm; 0005634: nucleus; 0000421: autophagosome membrane; 0005622: intracellular; 0031093: platelet alpha granule lumen. (D) GO analysis (molecular function) of 17 genes. 0005515: protein binding; 0070063: RNA polymerase binding; 0000976: transcription regulatory region sequence-specific DNA binding. (E) Kyoto Encyclopedia of Genes and Genomes analysis of 17 genes. hsa04140: Autophagy; hsa05022: Pathways of neurodegeneration; hsa05230: Central carbon metabolism in cancer; hsa04137: Mitophagy; hsa04210: Apoptosis; hsa04371: Apelin signaling pathway.

Based on the DE mRNAs, PPI network was constructed and visualized in Figure 1 A , and then top 30 hubgenes were selected, as shown in Figure 1 B . To identify the DE genes that were related to ferroptosis, DE mRNAs and ferroptosis gene list were intersected to obtain 17 genes as shown in Figure 2 A . And then GO enrichment analysis (BP,CC,MF) and KEGG pathway analysis were performed for the 17 genes, as shown in Figure 2 B, C, D, E  respectively. Subsequently, top 30 hubgenes and 17 DE ferroptosis genes were intersected to obtain 5 core ferroptosis hubgenes, as shown in Figure 1 C, D . 

As shown in Figure 2, ALB may locate in nucleus and platelet alpha granule lumen, participate in the biological process of negative regulation of programmed cell death and cellular response to starvation. KRAS may participate in negative regulation of cell differentiation, and implicate in several pathways, as shown in Figure 2 E . MAP1LC3A may locate in autophagosome membrane, participate in the biological process of cellular response to amino acid starvation and cellular response to starvation, and implicate in several pathways, as shown in Figure 2 E . CTSB may locate in intracellular and perinuclear region of cytoplasm, and implicate in 2 pathways: autophagy and apoptosis. HNRNPL may perform the function of specific DNA binding, locate in nucleus, ribonucleoprotein granule and perinuclear region of cytoplasm, and participate in several biological process, as shown in Figure 2 B . 

### CeRNA network and TF-miRNA-mRNA network of the core genes

**Figure 3 -:**
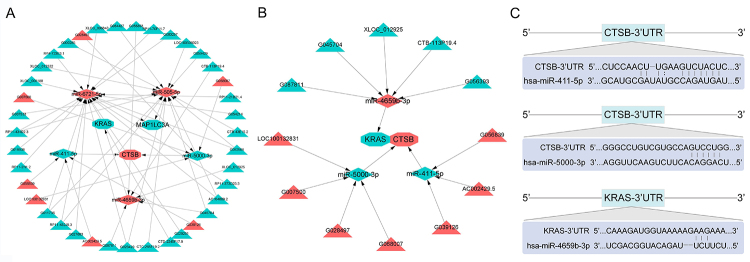
Competitive endogenous RNAs (ceRNAs) network and binding sites of mRNA-miRNA. (A) Preliminary ceRNA network. Triangles indicate lncRNAs, diamonds indicate miRNAs, and octagons indicate mRNAs. Red means upregulated; green means downregulated. (B) Simplified ceRNA network. The mRNA-miRNA with predicted binding sites were extracted from the preliminary ceRNA network. (C) The binding sites of mRNA-miRNA.

We constructed ceRNA network and TF-miRNA-mRNA network for five core genes. Regarding the preliminary ceRNA network, ALB and HNRNPL were not included, because of their targeting lncRNAs or miRNAs cannot intersect with DE lncRNAs or miRNAs. Accordingly, the preliminary ceRNA network is shown in Figure 3 A . To further verify the network, we predicted the binding sites for the mRNAs and their targeting miRNAs. Finally, the simplified ceRNA network is shown in Figure 3 B . And then the specific binding sites are visualized in Figure 3 C . 

**Figure 4 -:**
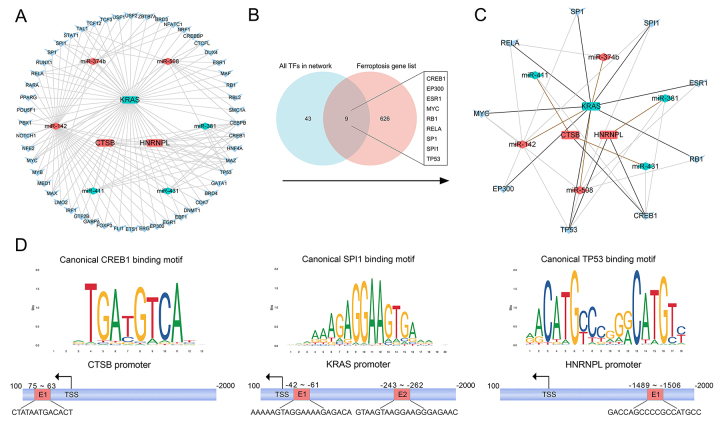
Transcription factor-miRNA-mRNA regulatory network and binding sites of transcription factor-mRNA. (A) Preliminary transcription factor-miRNA-mRNA network. TF: transcription factor; Round rectangles indicate mRNA, diamonds indicate miRNAs, and V-shapes indicate transcription factors. Red means upregulated; green means downregulated. (B) All the transcription factors in the network intersected with ferroptosis gene list to obtain 9 transcription factors s. (C) Simplified transcription factor-miRNA-mRNA network. The intersected 9 transcription factors and their targeted miRNAs and mRNAs were extracted from the preliminary network. (D) The binding sites of transcription factors-mRNA. TSS: transcription start site.

Regarding the preliminary TF-miRNA-mRNA network, ALB and MAP1LC3A were not included, because the targeting miRNAs cannot intersect with DE miRNAs, and targeting TFs cannot intersect with the TFs that predicted by the targeting miRNAs, respectively. Accordingly, preliminary TF-miRNA-mRNA network is shown in Figure 4 A . To simplify the network, we intersected all the TFs in the preliminary network with the ferroptosis gene list to obtain 9 TFs, as shown in Figure 4 B. Finally, the simplified network was shown in Figure 4 C . Based on the predicted binding score of mRNA-TF, the binding site of top1 TF and mRNA was visualized in Figure 4 D .

### Verification of the core genes via qRT-PCR

**Figure 5 -:**
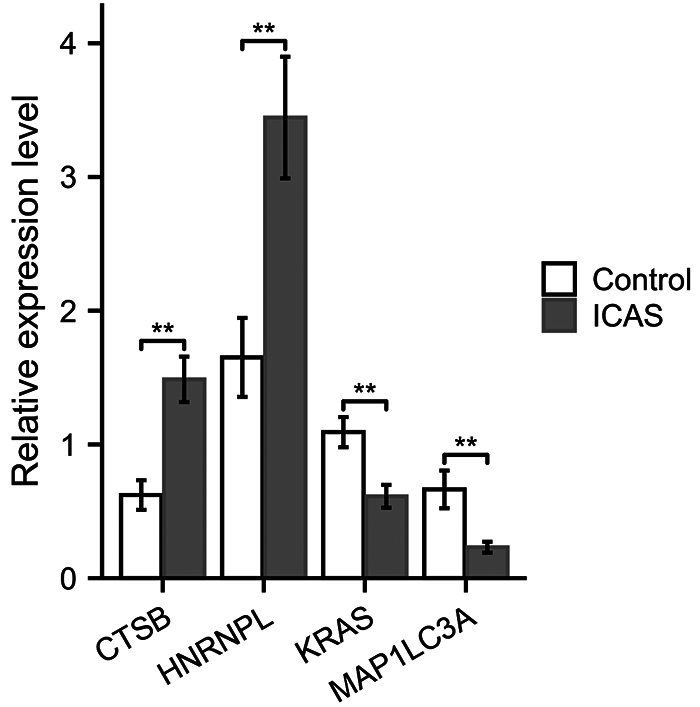
qRT-PCR verification of core genes. CTSB and HNRNPL were upregulated in the intracranial atherosclerotic stenosis group. KRAS and MAP1LC3A were downregulated in the intracranial atherosclerotic stenosis group. ICAS group: n=11, control group: n=9. ICAS: intracranial atherosclerotic stenosis. _**_: P<0.01.

The expression level of CTSB, HNRNPL, KRAS and MAP1LC3A were verified via qRT-PCR. As shown in the Figure 5, CTSB (P=0.001) and HNRNPL (P=0.002) were upregulated, KRAS (P=0.003) and MAP1LC3A (P=0.004) were downregulated. The qRT-PCR results of all the four mRNAs were consistent with the results of mRNA microarray expression profile.

## Discussion

Neutrophils are vital for the process of atherosclerosis. Previous studies have reported that elevated neutrophil counts may indicate the presence of ICAS-related occlusion (Li et al., 2020). Increased neutrophil counts may participate in the development and progression of atherosclerosis, contributing to carotid plaque destabilization. The baseline neutrophil-lymphocyte ratio of the healthy population correlates with the development of ICAS (Li *et al.*, 2020). Higher peripheral neutrophil counts correlate with a higher risk of ICAS in patients with AIS. Additionally, neutrophil count relates to the number and locations of ICAS (Zhang et al., 2020). Previous studies found that the neutrophil-to-lymphocyte ratio is positively related to intracranial atherosclerosis severity. Neutrophil counts in patients with large artery atherosclerosis stroke may increase with the number of craniocervical atherosclerotic stenoses (Kou et al., 2021). The increased neutrophil counts facilitate inflammatory responses by releasing proinflammatory factors (cytotoxins, reactive oxygen species (ROS), arachidonic acid metabolites, and proteolytic enzymes), causing endothelial cell dysfunction and accelerating atherosclerosis (Zhang *et al.*, 2020). Previous studies mainly focus on the relationship between neutrophil counts and clinical symptoms, or the interaction between neutrophils and other cells, such as smooth muscle cells and endothelial cells. However, the pathophysiological process of neutrophils themselves in atherosclerosis needs more attention. To date, no literature is available regarding the role of neutrophil ferroptosis in atherosclerosis. This is the main purpose of our study.

Our results detected four core genes which may participate in the pathophysiological processes of ICAS via regulating the ferroptosis of neutrophils. Both the results of mRNA microarrays and qRT-PCR verification show that CTSB and HNRNPL were upregulated, and KRAS and MAP1LC3A were downregulated in the circulating neutrophils of the patients with ICAS. Regarding the four core genes, we constructed ceRNA network and TF-miRNA-mRNA network to explore the potential regulatory network and upstream targets. Additionally, the potential binding sites of miRNA-mRNA and TF-mRNA were predicted and visualized. Our results focus on exploring the role of neutrophil ferroptosis in ICAS, providing insights for the pathophysiological processes of ICAS. 

KRAS is known as a small guanine-nucleotide binding protein, participating in the signal transduction related to cells growth regulation (Gillette et al., 2015). KRAS can induce smooth muscle proliferation and migration, participating in the process of atherosclerosis (Chen et al., 2022). While the literature about KRAS and neutrophil in atherosclerosis are not available. Cells expressing near-endogenous levels of oncogenic KRAS present an absence of ferroptosis-induced lipid peroxidation and an unregulated level of ferroptosis suppressor protein 1 (FSP1). Increased FSP1 can mediate ferroptosis resistance (Müller et al., 2022). In our study, KRAS was downregulated, which suggests that ferroptosis may be elevated. The ceRNA network show that KRAS may be regulated by several lncRNAs via miR-4659b-3p. To date, no literature is available to the relationship between miR-4659b-3p and ferroptosis. The TF-miRNA-mRNA network show that KRAS may be regulated by eight TFs (EP300, ESR1, MYC, RB1, RELA, SP1, SPI1, TP53) via miR-142, miR-374b and miR-508. Among the eigth TFs, the score of SPI1 conjugating with KRAS is highest. SPI1 is also known as PU.1, which is vital for development of myeloid and lymphoid lineages cells (Zakrzewska et al., 2010). SPI1 disruption correlates with defects in neutrophils (McKercher et al., 1996). SPI1 can indirectly inhibit radiotherapy-induced ferroptosis of esophageal squamous cell carcinoma cells (Zhao et al., 2023). Familial hypercholesterolemia (FH) is characterized by high serum level of low-density lipoprotein cholesterol, which can accelerate atherosclerosis. SPI1 is vital in the familial hypercholesterolemia related regulatory network (Chen *et al*., 2014). As shown in the TF-miRNA-mRNA network, SPI1 regulates KRAS via miR-142. According to literature, miR-142-3p is essential in the development and maturation process of neutrophil (Fan et al., 2014). Exosomal miR-142-3p of hepatocellular carcinoma cells promotes M1-type macrophage ferroptosis (Hu et al., 2022). Consistently, in our study, KRAS was downregulated and miR-142 was upregulated, which suggests that ferroptosis may be elevated. Regarding the interaction between SPI1 and KRAS, previous studies have verified their interaction in myeloid leukemia and radiotherapy-associated acute myeloid leukemia (Melo-Cardenas et al., 2018; O’Brien et al., 2020). Knocking out miR-142 can increase KRAS expression in the lung tissue of mice (Shrestha et al., 2019). The interaction pattern is consistent with our results.

Cathepsin-B (CTSB) is one of the lysosomal cysteine proteases family, involved in intracellular proteolysis. Additionally, CTSB may exert different effects in regulating cellular functions varying by substratum, cell type and other factors (Ruan et al., 2016). Studies have reported that CTSB is essential for albumin breakdown, accordingly affects the process of glutathione synthesis, lethal lipid peroxidation and ferroptosis (Armenta et al., 2022). Nuclear CTSB can activate the DNA sensor pathway and degrade GPX4, inducing autophagy-dependent ferroptosis (Kuang et al., 2020). CTSB can cause the cellular membrane destruction and chromatin degradation, causing ferroptotic cell death (Nagakannan et al., 2021). These studies suggest that CTSB may promote ferroptosis. Our results show that CTSB was upregulated in the circulating neutrophils of the patients with ICAS. Combined with the results and literature, we can deduce that neutrophil ferroptosis may contribute to the pathophysiological processes of ICAS. The ceRNA network shows that CTSB may be regulated by several lncRNAs via miR-411-5p and miR-5000-3p. No literature was available to the interaction between CTSB and miR-411-5p/ miR-5000-3p. The TF-miRNA-mRNA network show that CTSB may be regulated by CREB1 via miR-411 and miR-431. In our study, CTSB was upregulated and miR-431 was downregulated. Combined with the inference mentioned above, we can further deduce that miR-431 may inhibit ferroptosis. Consistently, literature has reported that upregulated miR-431 inhibit nucleus pulposus cells ferroptosis via circ_0072464/miR-431/NRF2 axis (Yu et al., 2022b). While the literature about the relationship between miR-411 and ferroptosis was not available. Cyclic adenosine monophosphate (cAMP) responsive element binding protein 1(CREB1) is known as a transcription factor which bind to promoter cis-regulatory elements, ubiquitously expressing in many tissues (Li et al., 2022). CREB can promote the transcription of GPX4 in the absence of EP300, suppressing ferroptosis in lung adenocarcinoma (Wang et al., 2021 b ). While the relationship between CREB1 and CTSB has not been reported. Our study firstly proposes that CREB1 may regulate CTSB via miR-431 and miR-411, modulating neutrophil ferroptosis and participating in the process of ICAS. Previous studies have identified the potential interaction between CREB1 and CTSB (Omori et al., 2007; Zhao et al., 2024). However, the interaction between CTSB and miR-431, miR-411, and miR-5000-3p has not been verified.

Heterogeneous nuclear ribonucleoprotein L (HNRNPL) is a type of RNA binding protein (RBP), mainly locating in the nucleus. HNRNPL can exert various effects, such as DNA repair, protein translation and RNA alternative splicing (Gu et al., 2020). RBPs usually exert effects depending on conjugating with mRNAs, RBPs, or non-coding RNAs, other than act alone (ME, 2022). Inhibition of HNRNPL can destabilize YY1 mRNA and enhance T cell-mediated cancer cell ferroptosis in castration-resistant prostate cancer (Zhou et al., 2022). The TF-miRNA-mRNA network shows that HNRNPL may be regulated by CREB1/TP53 via miR-381. The context of CREB1 has been discussed in the last paragraph. The relationship between CREB1 and HNRNPL has not been reported. The tumor suppressor p53 (TP53) blocks the activity of dipeptidyl-peptidase-4 (DPP4), antagonizing erastin-induced ferroptosis in human colorectal cancer in a transcription-independent manner (Xie et al., 2017). While other studies have reported that TP53 may serve as a positive regulator of ferroptosis (Wang et al., 2016). HNRNPL indirectly inhibits p53 in mouse embryonic stem cells (Li et al., 2015). Increased miR-381 inhibits EGR1/p53 to exert neuroprotective effect in mice postoperative cognitive dysfunction model (Wang *et al.*, 2021a). In our study, HNRNPL was upregulated and miR-381 was downregulated. Given that HNRNPL belongs to RBPs, acting depend on conjugating with other factors, and the bidirectional control of ferroptosis by p53 and the regulation of HNRNPL/ miR-381 and p53, the expression level of HNRNPL is unreliable to deduce the status of ferroptosis in ICAS group. Further studies are needed to explore the exact regulatory network between these factors. Regarding the interaction between HNRNPL and the molecules studied, there is no literature available to date. 

Microtubule-associated protein light chain 3 (MAP1LC3A) is also known as LC3A (Cheng et al., 2016). LC3 family of human consists of 3 members: LC3A, LC3B, and LC3C. LC3B is known as a marker for autophagy, while the relationship between autophagy and LC3A/ LC3C remain unclear (Baeken et al., 2020; Fan et al., 2021). Ferroptosis is a type of cell death depending on autophagy (Xue et al., 2023). According to literature, inhibition of LC3 I/II can alleviate autophagy-induced ferroptosis in transient middle cerebral artery occlusion model (Zhu et al., 2022). In our study, the ceRNA network shows that MAP1LC3A may be regulated by several lncRNAs via miR-505-5p and miR-6721-5p. Our results show that MAP1LC3A was downregulated, and miR-505-5p and miR-6721-5p were upregulated. Consistently, literature suggest that increased miR-505 enhances reactive oxygen species level and neutrophil extracellular traps formation in neutrophil, aggravating atherosclerosis (Chen *et al*., 2019). Ferroptosis depends on high levels of intracellular oxidative stress induced by reactive oxygen species (ROS) accumulation (Yu et al., 2022 a ). Hsa-miR-6721-5p is involved in the oxidative stress injury of melanocytes (Li et al., 2021). We deduce that miR-6721-5p may be implicated in ferroptosis. Previous studies have found that miR-505 significantly inhibits autophagy in neuronal cells after Borna disease virus 1 infection. Overexpression of miR-505 decreases the autophagy-related marker LC3 (Guo et al., 2022). The interaction pattern is consistent with our results. To date, no literature is available on the interaction between MAP1LC3A and miR-6721-5p. Further studies are needed.

There were several limitations in our study. Firstly, the small sample size has to be considered. Secondly, given the regulatory networks were constructed by computational prediction, the limitations of computational predictions should be acknowledged. Further molecular biology experiments are needed to verify the role of neutrophil ferroptosis in ICAS and to clarify the potential interactions between these key molecules.

## Conclusion

In summary, our results suggest that neutrophil ferroptosis may participate in the pathophysiological process of ICAS. We identified four core genes (CTSB, HNRNPL, KRAS and MAP1LC3A) and constructed potential regulatory network, which may provide the potential therapeutic targets for ICAS and pave the way for further research.

## Supplementary Material

The following online material is available for this article:

Table S1 - The primer sequences of mRNAs.

Figure S1 - Flow chart.

Figure S2 - Volcano plots and heatmaps.

## Data Availability

All data generated or analyzed during current study are available from the corresponding author on reasonable request.
