# Supplementary material for: Identification and interaction analysis of hubgenes related to neutrophil ferroptosis in intracranial atherosclerotic stenosis
Source: Genet Mol Biol. 2025 Nov 17;48(3):e20240106. doi: 10.1590/1678-4685-GMB-2024-0106 (PMC12629527; doi:10.1590/1678-4685-GMB-2024-0106)
Supplement: Table S1 - [file 1415-4757-GMB-48-3-e20240106-s1.pdf]

Genetics and Molecular Biology
https://doi.org/10.1590/1678-4685-GMB-2024-0106

Supplementary Material to "Identification and interaction analysis of hubgenes related to neutrophil ferroptosis in intracranial atherosclerotic stenosis"

Table S1 - The primer sequences of mRNAs.

Gene name	Primer Sequence	Temperature (◦C)	Product Length (bp)
β-actin	F:5' GTGGCCGAGGACTTTGATTG3'<br>R:5’ CCTGTAACAACGCATCTCATATT3’	60	73
CTSB	F:5' CAAACAGGACAAGCACTACGGA 3’<br>R:5’ GAGCAGGAAGTCCGAATACACA 3’	60	127
HNRNPL	F:5'GGAAGAATGGAGTTCAGGCG3’<br>R:5’ AGCGTGTAGGCTTTGCGTAT 3’	60	135
KRAS	F:5'GCTTTCTTTGTGTATTTGCCAT3’<br>R:5’ AGTCCTGAGCCTGTTTTGTGT3’	60	168
MAP1LC3A	F:5' AACATGAGCGAGTTGGTCAAGA 3’<br>R:5’ TCTCCTGCTCGTAGATGTCCG 3’	60	133
