# Supplementary material for: Identification and interaction analysis of hubgenes related to neutrophil ferroptosis in intracranial atherosclerotic stenosis
Source: Genet Mol Biol. 2025 Nov 17;48(3):e20240106. doi: 10.1590/1678-4685-GMB-2024-0106 (PMC12629527; doi:10.1590/1678-4685-GMB-2024-0106)
Supplement: Figure S1 - [file 1415-4757-GMB-48-3-e20240106-s2.pdf]

Genetics and Molecular Biology
https://doi.org/10.1590/1678-4685-GMB-2024-0106

Supplementary Material to "Identification and interaction analysis of hubgenes related to neutrophil ferroptosis in intracranial atherosclerotic stenosis"

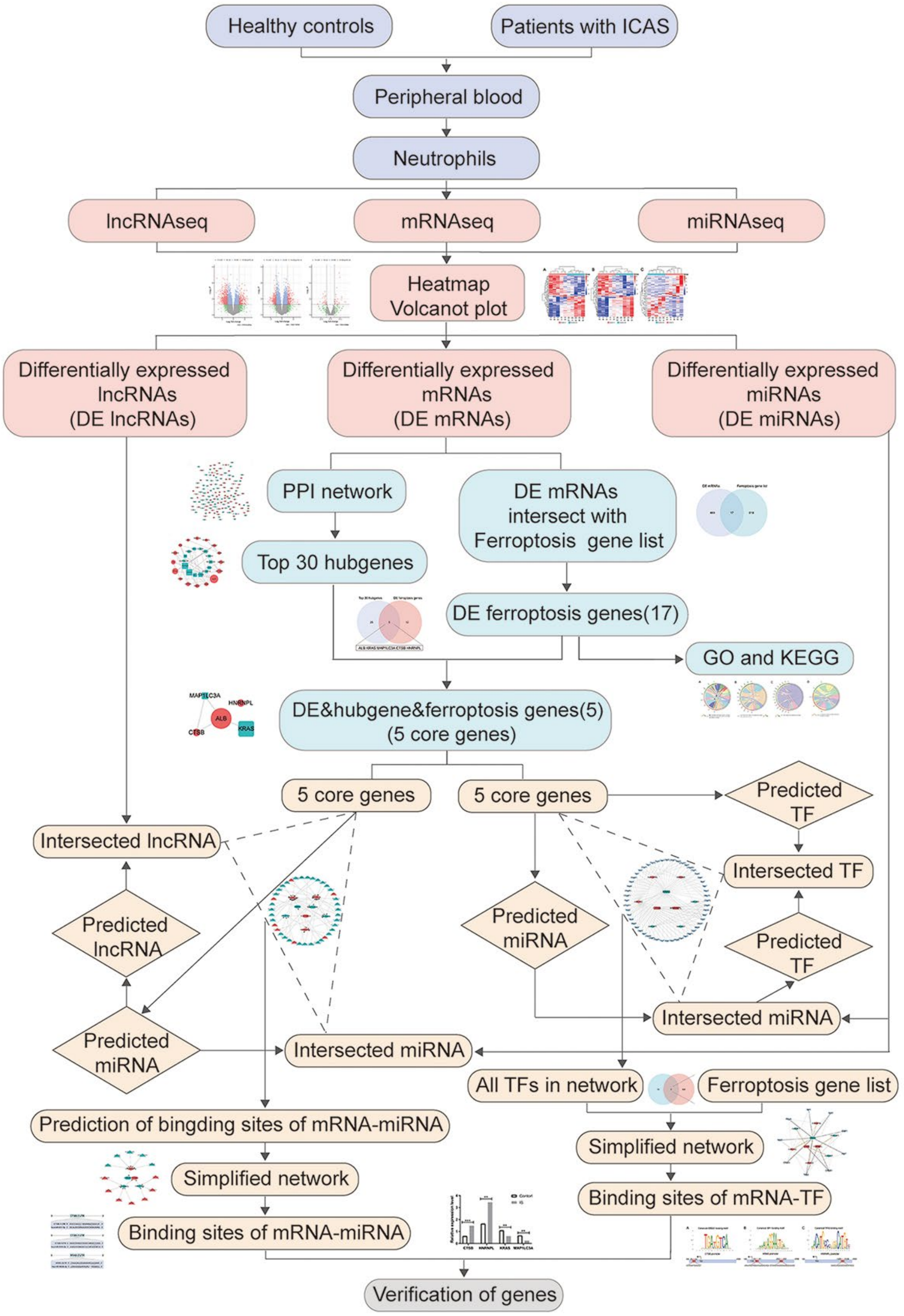

Figure S1 - Flow chart. ICAS: intracranial atherosclerotic stenosis, PPI: protein-protein interaction, GO: Gene Ontology, KEGG: Kyoto Encyclopedia of Genes and Genomes, TF: transcription factor.
