# Supplementary material for: Identification and interaction analysis of hubgenes related to neutrophil ferroptosis in intracranial atherosclerotic stenosis
Source: Genet Mol Biol. 2025 Nov 17;48(3):e20240106. doi: 10.1590/1678-4685-GMB-2024-0106 (PMC12629527; doi:10.1590/1678-4685-GMB-2024-0106)
Supplement: Figure S2 - [file 1415-4757-GMB-48-3-e20240106-s3.pdf]

Genetics and Molecular Biology
https://doi.org/10.1590/1678-4685-GMB-2024-0106

Supplementary Material to "Identification and interaction analysis of hubgenes related to neutrophil ferroptosis in intracranial atherosclerotic stenosis"

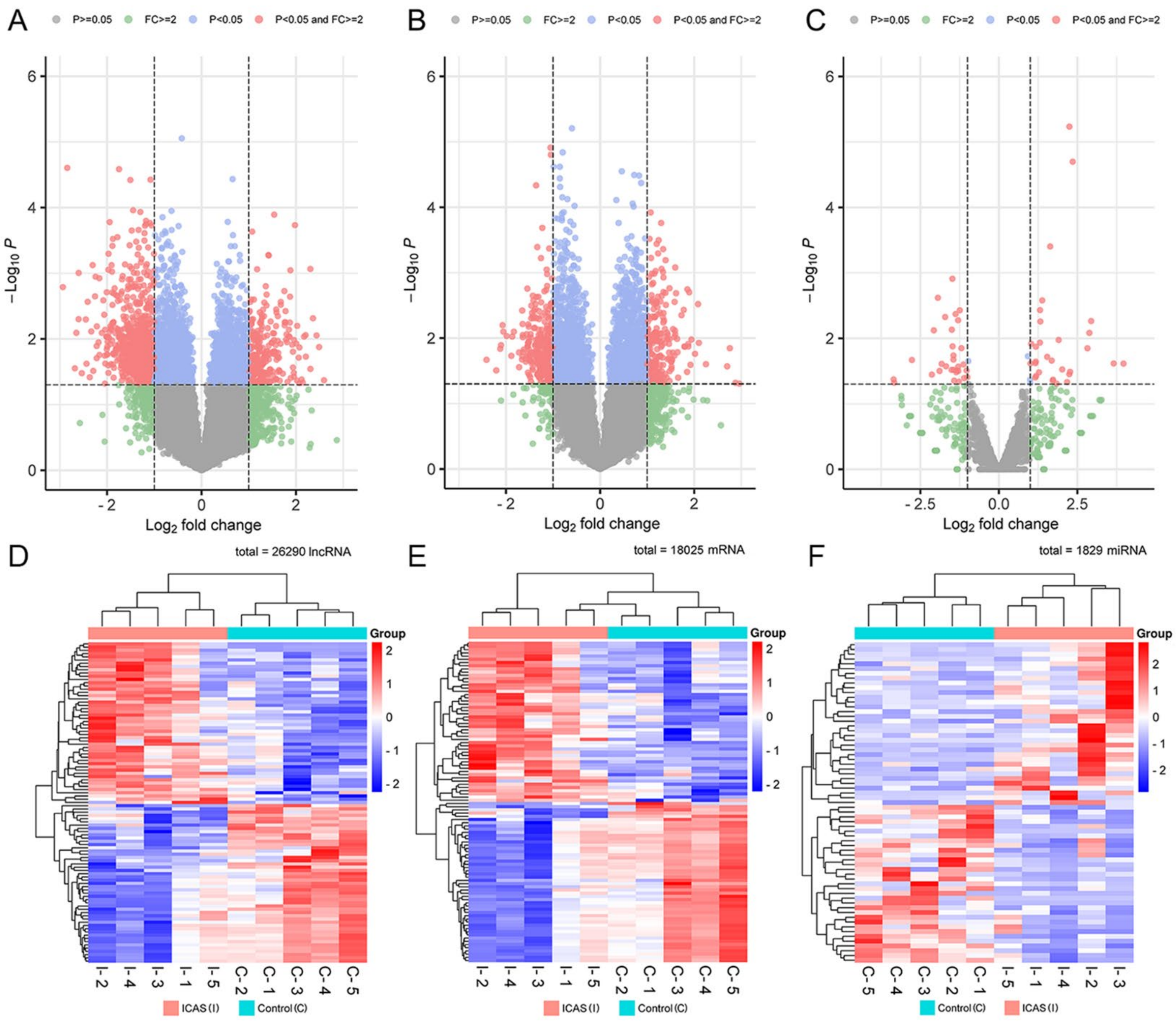

Figure S2 - Volcano plots and heatmaps. ICAS: intracranial atherosclerotic stenosis; FC: fold change. (A-C) display the Volcano plot of lncRNAs, mRNAs, and miRNAs, respectively. (D-F) display the heatmap of lncRNAs, mRNAs, and miRNAs, respectively.
